# HtrA1: Its future potential as a novel biomarker for cancer

**DOI:** 10.3892/or.2015.4016

**Published:** 2015-05-28

**Authors:** EMMA ALTOBELLI, DANIELA MARZIONI, AMEDEO LATTANZI, PAOLO MATTEO ANGELETTI

**Affiliations:** 1 Department of Life, Health and Environmental Sciences, Epidemiology and Biostatistics Unit, AUSL Teramo, University of L’Aquila, L’Aquila, Italy; 2 Department of Experimental and Clinical Medicine, University of Ancona, Ancona, Italy; 3 Department of Life, Health and Environmental Sciences, University of L’Aquila, L’Aquila, Italy

**Keywords:** HtrA1, human, diagnostic/prevention biomarkers

## Abstract

HtrA1 appears to be involved in several physiological processes as well as in the pathogenesis of conditions such as Alzheimer’s disease and osteoarthritis. It has also been hypothesized to play a role as a tumor suppressor. This manuscript reviews the current cancer-related HtrA1 research from the methodological and clinical standpoints including studies regarding its potential role as a tumor marker and/or prognostic factor. PRISMA method was used for study selection. The articles thus collected were examined and selected by two independent reviewers; any disagreement was resolved by a methodologist. A laboratory researcher reviewed the methods and laboratory techniques. Fifteen studies met the inclusion criteria and concerned the following cancer sites: the nervous system, bladder, breast, esophagus, stomach, liver, endometrium, thyroid, ovaries, pleura, lung and skin. Most articles described *in vivo* studies using a morphological approach and immunohistochemistry, whereas protein expression was quantified as staining intensity scored by two raters. Often the results were not comparable due to the different rating scales and study design. Current research on HtrA1 does not conclusively support its role as a tumor suppressor.

## Introduction

HtrA1, first described by Zumbrunn and Trueb (1), is expressed in different normal human tissues (2) and appears to be involved in several physiological processes, through inhibition of extracellular protein transforming growth factor β (TGF-β) signaling *in vivo* and *in vitro* (3), as well as in the pathogenesis of diseases such as amyloid degeneration, senile macular degeneration, Alzheimer’s disease, osteoarthritis and pre-eclampsia (4–7). It has also been hypothesized to play a role as a tumor suppressor. The first clinical study was carried out on melanoma by Baldi and colleagues (8), who found significant HtrA1 upregulation in the primary tumor compared with metastases, and suggested that HtrA1 expression could be an indicator of disease progression. The hypothesis has subsequently been tested in other neoplasms. A possible role for HtrA1 as a prognostic factor for cancer has also been hypothesized. Downregulation of HtrA1 protein is associated with poor survival in mesothelioma (9), hepatocellular carcinoma (10) and breast cancer (11); in the latter study node-positivity was associated with shorter survival. HtrA1 downregulation has also been observed to be associated with poor chemotherapy response in patients with gastric cancer (12). These findings suggest a possible prognostic role for HtrA1 expression. The present manuscript reviews current cancer-related HtrA1 research from the methodological and clinical standpoints and studies exploring the potential role of HtrA1 as a tumor marker and/or prognostic factor in a number of tumors.

## Materials and methods

A MEDLINE search was conducted in August 2014 using the terms: HtrA1 OR PRSS11 protein, human OR L56 protein, human OR protease, serine, 11 (IGF binding) protein, human OR high-temperature requirement factor A1, human OR HtrA serine peptidase 1, human AND Neoplasm OR Tumors OR Tumor OR Neoplasia OR Cancer OR Cancers. No restriction was applied in terms of date, language or study design. The PRISMA method was used to select studies (14). A search of Embase and the Cochrane Systematic Review and Clinical Trial Register did not yield additional studies. A manual search was also conducted. The articles thus collected were examined and selected by two independent reviewers (P.M.A. and A.L.); any disagreement was resolved by a methodologist (E.A.). The methods and laboratory techniques were reviewed by a laboratory researcher (D.M.).

Studies involving clinical studies and those where HtrA1 was determined either as mRNA or as protein were included; articles involving exclusively *in vitro* studies and exclusively animal studies, and the methods applied to detect HtrA1 in each study: *in situ* hybridization (ISH) polymerase chain reaction (PCR) immunohistochemistry (IHC) and western blotting (WB) were determined.

Those published as congress proceedings were excluded. Data are presented as tables. Table I documents the selected studies including the date of publication, sample size, age, gender and general characteristics of the patient population; HtrA1 values in the tumor and control tissue, and any association found between HtrA1 level, histological differentiation and TNM and/or clinical stage.

Table II shows the longitudinal observational data relating HtrA1 to survival.

Table III reports the methods applied to detect HtrA1 in each study: *in situ* hybridization (ISH), polymerase chain reaction (PCR), immunohistochemistry (IHC) and western blotting (WB).

Table IV reviews the current use of HtrA1 as a marker in oncology.

## Results

**Figure 1:**
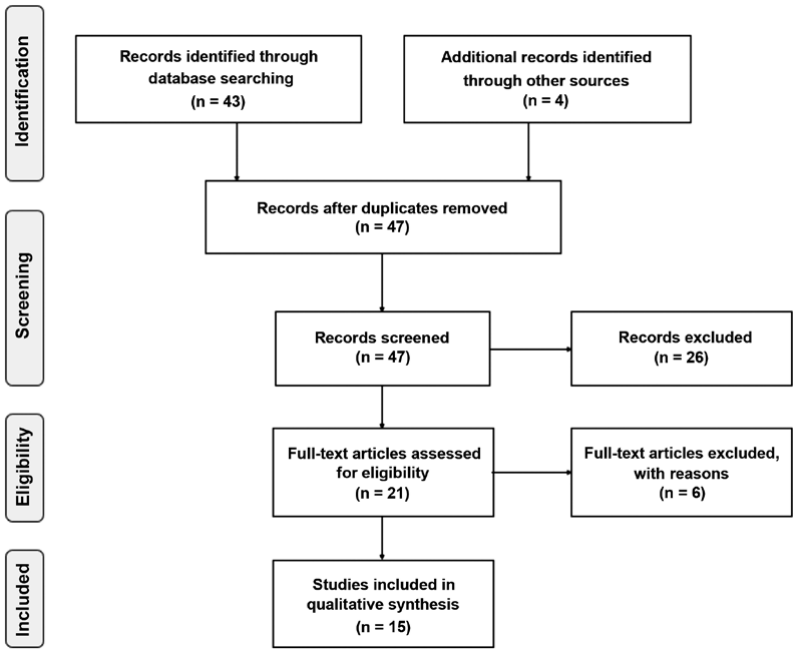
Flow-chart of the search strategy: HtrA1 and cancer.

The database search conducted as described above retrieved 43 studies, and the manual search retrieved 4 additional studies. No duplicates were found (Fig. 1). Examination of the abstracts led to the exclusion of 26 studies; 1 regarded rheumatoid arthritis (15); 3 studied macular degeneration (16–18); 2 investigated the placenta (7,19); 1 addressed tuberous sclerosis complex 2 (20); 3 were animal studies (21–23); 1 assessed amyloid precursor protein (4); 2 evaluated other biomarkers (24,25); 6 were exclusively *in vitro* studies (1;26–30); and 7 were reviews (2,31–36), thus leaving 21 studies. Examination of their full text led to the exclusion of 6 further studies since they did not report clinical studies (13,37–41). Overall, 15 studies met the inclusion criteria and are reviewed below.

### Synthesis of the data of observational studies and overview of laboratory techniques

The 15 studies included in the review describe recent HtrA1 research in relation to cancer.

### Neuroblastoma

There was a single investigation addressing neural tissue tumors, i.e. neuroblastoma (NB) and ganglioneuroblastoma (42). This is also the sole study involving a pediatric population. Its aim was to test the value of HtrA1 as a new biomarker of tumor differentiation and aggressiveness by quantifying its expression and localization in NB. D’Angelo *et al* (42) assessed HtrA1 expression by semi-quantitative methods: by IHC using the HSCORE value and by WB using Quantity One software, neither of which provides an absolute value of protein concentration. The authors examined 60 NB: 26 stage I-II; 14 stage III; 16 stage IV; and 4 stage IV tumors. The statistical distribution of the HtrA1 variable was not investigated. Higher HtrA1 protein levels were found in stages associated with a more favorable prognosis, but their possible correlation with gender or age was not assessed.

### Bladder cancer

The single study of bladder cancer by Lorenzi and co-workers (43) examined HtrA1 expression in tissue and urine from 152 subjects, 38 healthy individuals, 68 patients with urothelial cancer, and 16 subjects with cystitis, to establish a possible association with urothelial cancer. Two forms of HtrA1, a 50- and a 38-kDa autocatalytic form, were detected in tissue specimens. The autocatalytic form was downregulated in cancer tissue whereas significantly higher levels of both forms were measured in urine from cancer patients compared with healthy individuals. HtrA1 distribution was normal in urine from all participants. The correlation of HtrA1 levels with gender and age was not tested. Global test performance was assessed. The authors applied molecular, morphological and biochemical techniques. Since mRNA and protein levels were not measured in the whole sample, but only in radical cystectomy tissue, the results apply only to this group.

### Breast cancer

Lehner and co-workers (11) assessed the impact of HtrA1 mRNA expression on patient outcome in a cohort of 131 cancer patients using molecular and *in vitro* techniques to measure HtrA1 mRNA and HtrA1 promoter hypermethylation, the latter as an HtrA1 silencing mechanism in breast tumors. HtrA1 protein expression was not assessed. The statistical distribution of the HtrA1 variable was not investigated. Significantly higher HtrA1 expression was found in lower tumor stages, but no relationship was found with grade (Table I). There were no significant differences related to the expression of progesterone and estrogen receptor; pre- or post-menopausal state; or type of surgical management (lumpectomy vs. mastectomy). High levels of HtrA1 mRNA were associated with longer overall survival (OS) and disease-free survival (DFS) (Table II).

### Esophageal cancer

Yu and colleagues (44) explored the possible involvement of HtrA1 levels in cell invasiveness, differentiation, stage and metastasis formation in esophageal cancer tissue and adjacent normal-appearing tissue from 63 patients. HtrA1 mRNA and protein were measured using semi-quantitative biomolecular methods. The statistical distribution of the HtrA1 variable was not examined. Significantly higher HtrA1 mRNA (P=0.004) and protein (P<0.05) levels were detected in normal-appearing tissue. The authors found that HtrA1 mRNA and protein levels were higher in well and moderately differentiated tumors than in poorly differentiated tumors (respectively, mRNA P=0.024; protein P<0.05) as well as in early compared with late disease stages (I-II vs. III-IV, respectively, mRNA P=0.013; protein P<0.05). The data regarding metastases are particularly important, since HtrA1 mRNA and protein levels were lower in tumors with distant metastases that in those without metastases (mRNA P<0.001; protein P<0.05). Similar data were found for lymph node metastases (mRNA P=0.002; protein P<0.05). In contrast HtrA1 mRNA was not found to correlate with gender, age or size of the primary tumor.

Xia and co-workers (45) evaluated HtrA1 mRNA and protein in cancer and normal-appearing tissue using respectively IHC and ISH, two semi-quantitative techniques that were mainly applied to detect the cell type expressing HtrA1. Staining (positive or negative) was assessed in a blinded manner by two independent researchers. *In vitro* analysis was used to detect the molecular targets of HtrA1. The sample consisted of 115 patients with squamous cell carcinoma (SCC) of the esophagus. The statistical distribution of the HtrA1 variable was not examined. The authors found an association between HtrA1 level and TNM stage (Table I), but not between tumor differentiation, gender or age. Mean follow-up duration was 40 months. HtrA1 mRNA and protein levels correlated with survival rate, since HtrA1 (mRNA and protein)-positive patients had a longer survival than HtrA1-negative subjects (Table II).

### Gastric cancer

Catalano and colleagues (12) mainly assessed whether HtrA1 levels affect chemo-responsiveness in 80 patients with advanced gastric cancer. They used only morphological analysis to evaluate HtrA1 expression in diseased tissue. Staining was evaluated by three raters using an observational semi-quantitative method that does not measure absolute protein value, but the change in staining intensity. The statistical distribution of the HtrA1 variable was not examined. HtrA1 expression was associated with chemo-responsiveness, and higher HtrA1 levels correlated with longer survival and time to progression. There were no associations with gender, age or Lauren’s classification.

### Hepatocellular carcinoma

Zhu *et al* (10) measured HtrA1 expression in liver tissue from 50 patients with hepatocellular carcinoma to establish whether it may have a role in cancer development and progression. HtrA1 was assessed by IHC; staining intensity was evaluated by light microscopy using a semi-quantitative score. HtrA1 levels correlated with Edmondson and Steiner’s criteria and vascular invasion (P=0.014) but not with gender, age, tumor size or presence of metastases. The statistical distribution of the HtrA1 variable was not examined. After a follow-up of 3 years, the survival was longer in the HtrA1-positive (46%) than in the HtrA1-negative patients (26%).

### Endometrial cancer

Mullany and co-workers (46) investigated by an *in vitro* model whether HtrA1 downregulation influences cancer cell invasion. In addition, randomly selected patients from a historical cohort of 184 patients were used for IHC. Of these, 142 had tumor stages I-II and 42 had stages III-IV; 65 had histological grade 1, 75 histological grade 2, and 44 histological grade 3; 171 had endometroid or mucinous disease and 13 had non-endometroid cancer. The authors did not examine the whole section, but used a micro-array technique to analyze 3 endometrial samples 0.6-mm in diameter from each patient. HtrA1 expression was evaluated as staining intensity. The statistical distribution of the HtrA1 variable was not investigated. HtrA1 levels were lower in patients with less differentiated tumors, whereas they did not correlate with clinical stage, myometrial invasion, age or survival.

Narkiewicz and colleagues (47) studied HtrA1, HtrA2 and HtrA3 mRNA and protein by two semi-quantitative techniques, RT-PCR (mRNA) and WB (protein), in 124 women; 88 with endometrial cancer and 36 with normal endometrium. The statistical distribution of the HtrA1 variable was not examined. They documented significant differences in HtrA1 expression between normal and cancer tissue, whereas differences in FIGO stage, histological type or grade or menopausal state were not significant.

Bowden and colleagues (48) assessed HtrA1 and HtrA3 mRNA and protein by semi-quantitative PCR, IHC and WB in 33 women. HtrA1 mRNA was significantly reduced in tumors compared with normal tissue, whereas protein levels were not significantly different. Neither HtrA1 distribution nor its relationship with age or menopausal state were explored.

### Thyroid cancer

Zurawa-Janicka *et al* (49) assessed HtrA1 in tissue extracts from 40 subjects; 20 with normal thyroid and 20 with follicular (n=12) or papillary (n=8) tumors using WB. Protein band intensities assessed by densitometry yielded relative values. There were no differences between healthy and cancer tissue, whereas slightly higher HtrA1 protein levels were found in follicular than in papillary cancer (P=0.045). Neither HtrA1 distribution nor its relationship with age or gender were examined.

### Ovarian cancer

Narkiewicz and co-workers (50) assessed HtrA1 mRNA and protein in 98 women with various types of ovarian tumors (20 benign, 7 borderline, 44 malignant, and 8 Krukenberg tumors) or with healthy ovaries (n=19) using densitometry, which provided relative values of HtrA1 expression. Significant mRNA downregulation was found in malignant tumors (P<0.001), whereas the protein level did not exhibit significant differences. Histological type and grade did not display significant differences. Neither HtrA1 distribution nor its relationship with age were examined.

### Pleural mesothelioma

Baldi *et al* (9) examined the prognostic role of HtrA1 and EGFR (epidermal growth factor receptor) in 70 mesotheliomas whose histological type was epithelioid (n=45), mixed (n=14) and sarcomatoid (n=11). Their T status was T1 (n=4); T2 (n=13); T3 (n=23); T4 (n=4), and TX (n=26); their N status was N0 (n=27); N1 (n=3); N2 (n=14); and NX (n=26). The authors investigated HtrA1 expression by IHC and measured its level by an observational semi-quantitative method. The statistical distribution of the HtrA1 variable was not examined. HtrA1 was not assessed in relation to gender or age. Findings showed that the HtrA1 level was closely related to survival, highlighting a role for HtrA1 as a marker, especially for prognosis.

### Lung cancer

The study by Esposito and colleagues (51) evaluated HtrA1 expression in 99 patients with primary lung tumors and metastasis using IHC. The intensity of HtrA1 staining was evaluated by an observational method using a score. There were 43 patients with SCC; 45 with adenocarcinoma and 11 with other histological types. Clinical stage was I in 12 patients II in 34, IIIa in 27, and IIIB in 26 patients. Management was surgical in 72 and non-surgical in 27 patients. The statistical distribution of the HtrA1 variable was not investigated. The authors found that HtrA1 was underexpressed in metastases compared with the primary tumor. Differences regarding histological type, TNM or stage were not significant. HtrA1 was not assessed in relation to gender or age.

### Malignant melanoma

Baldi and co-workers (8) investigated HtrA1 expression by IHC using tissue array. Staining intensity was evaluated by an observational semi-quantitative approach. This was the first study reporting clinical data of patients with malignant melanoma and demonstrated higher HtrA1 levels in primary tumors compared with metastases. The statistical distribution of HtrA1 was not tested.

The laboratory approaches applied to detect HtrA1 in a variety of tumors are reported in Table III. Most articles described *in vivo* studies using a morphological approach and IHC, whereas protein expression was quantified as staining intensity scored by two raters. Often the results were not comparable due to the different rating scales. WB was used to detect HtrA1 protein both in tissue and cell extracts. Protein band intensity was assessed by densitometry using two different but comparable softwares, Quantity One (Bio-Rad Laboratories, Hercules, CA, USA) and 1Dscan EX 3.0 (Scanalytics, Rockville, MD, USA). *In vitro* analysis was performed mainly to explore the role of HtrA1 in cell proliferation, migration and invasion. In addition, Baldi *et al* (8), Xia *et al* (45) and Lehner *et al* (11) investigated the molecules that may be involved in the regulation of HtrA1 activity. The data regarding HtrA1 as a potential biomarker in oncology are reported in Table IV.

## Discussion

A role for HtrA1 in cell proliferation has been described in a small number of studies (8,52,53) for conditions such as macular degeneration (52) and skeletal osteoarthritis (31). Its involvement in proliferation processes has suggested a possible role as a tumor suppressor. Even though HtrA1 has been investigated in a variety of tumors for more than a decade, the comparison of findings is hampered by differences in design as well as in the clinical and laboratory detection methods used. Such lack of uniformity is clearly apparent in the 15 studies reviewed above, most of which use mainly a semi-quantitative method of analysis that does not provide absolute values; this entails that any differences that are found can be compared only within each study. In addition, most studies use tissue samples from pathological files, which have been shown to be useful to diagnose early pathological changes, but are unsuitable for large-scale screening of high-risk populations. A broader clinical application of such approaches would have to rely on the technical ability of individual clinical pathology laboratories.

### HtrA1: a possible tissue biomarker

The present study highlighted a consistent finding that was reported by all of the studies that tested this aspect, i.e. that HtrA1 levels are higher in healthy or normal-looking control tissue than in diseased tissue from patients with a variety of tumors. The difference was statistically significant in urothelial bladder (43), esophageal (44,45), liver (10), and endometrial (47,48) cancer, but not in ovarian cancer (50). The remaining studies, which involved NB (42), breast (11), gastric (12), endometrial (46) and lung cancer (51), pleural mesothelioma (9) and malignant melanoma (8), did not investigate the issue.

The correlation of HtrA1 with histological differentiation was inconsistent, since it was demonstrated in esophageal (44) and endometrial (46,48) cancer and hepatocellular carcinoma (10), but not in breast (11), esophageal (45), and ovarian (50) cancer, whereas it was not assessed in NB (42), bladder (43), gastric (12), and endometrial cancer (47), pleural mesothelioma (9) and malignant melanoma (8).

Assessment of the relationship of HtrA1 with TNM and clinical stage also yielded conflicting data, since a clear relationship was found in NB (42) and esophageal cancer (44,45), but not in gastric (12), endometrial (46,47) ovarian (50), lung (51) or liver (10) tumors.

A correlation with tumor size was found only for breast cancer (11) and malignant melanoma (8). In the two clinical studies of malignant melanoma and lung carcinoma (8,51) analysis of HtrA1 levels in primary and metastatic tumors demonstrated lower levels in metastasis, suggesting a role for HtrA1 as a possible growth regulatory factor in the complex controlling cell growth in normal and transformed cells. Further investigation by a single study (45) failed to find significant differences. Additional studies are therefore required to clarify the role of HtrA1 in the growth of normal and tumor tissue cells.

The current literature does not, therefore, provide conclusive data on the role of HtrA1 as a tumor marker, but documents the need for further research.

### HtrA1:a possible diagnostic biomarker

A single study (43) assessed HtrA1 as a potential diagnostic marker. The study used ELISA, which provided a continuous numerical value of protein, and documented a good performance of HtrA1 in urothelial cancer diagnosis. Since these data were obtained in a small sample and have not been supported by other studies in biological fluids, they require validation in a larger sample.

### HtrA1: a possible prognostic biomarker

The present review found six longitudinal studies.

Catalano and colleagues (12) investigated the chemo-responsiveness of gastric cancer in relation to HtrA1 levels in terms of OS and DFS, hypothesizing that the HtrA1 level may be used as predictor of responsiveness to chemotherapy.

HtrA1 levels were also evaluated by Lehner *et al* (11) in breast cancer in relation to OS and DFS. They found that patients with higher HtrA1 levels had a better prognosis.

A longer OS was also measured in patients with pleural mesothelioma showing higher HtrA1 levels (9).

Finally, the investigation of liver (10) and esophageal (45) cancer also documented a longer OS in patients with a greater HtrA1 expression, whereas the difference found in endome-trial cancer was not significant (46). These data suggest that HtrA1 may be used as a predictor of OS.

### Research implications

The current HtrA1 research does not conclusively support its role as a tumor suppressor. Clinical investigations sharing similar approaches, especially in terms of study design, are needed to produce comparable data.

In the light of the findings reviewed above, HtrA1 research should focus on its role as a marker for early diagnosis in selected patients; notably, establishing a diagnostic gold standard would enable early diagnosis especially of tumors for which a screening test is not available. It would also be interesting to explore whether HtrA1 has a role in those tumors for which a screening test is available but has suboptimal sensitivity, such as colorectal cancer. Colorectal cancer ranks first in incidence and second in mortality in Europe for both genders (53), yet, HtrA1 has never been investigated as a possible diagnostic and/or prognostic marker in this tumor.

Recent advances in RNA sequencing, circulating DNA methylation profiling, and glycoproteins may allow the development of non-invasive diagnostic biomarkers for routine monitoring. Future studies should combine different classes of circulating biomarkers in large-scale investigations to improve the predictive power of the individual biomarkers. The development of assays for circulating biomarkers providing absolute and reproducible values would help conduct large-scale multi-center investigations and promote the use of circulating biomarkers in routine clinical practice.

## Figures and Tables

**Table I:** Characteristics of the 15 studies surveyed.

Author, year (ref.)	Cancer site	Patient population				HtrA1 expression		
		Sample size	Gender/Age (years)	Characteristics of the selected study	Clinical notice	Cancer tissue vs. control tissue	Degree of differentiation	Relationship with TNM or clinical stage
D’Angelo *et al*, 2014 (42)	Autonomic nervous system (neuroblastoma ganglioneuroblastoma)	60	Male, 34 Female, 26 Age <12, 17 Age >12, 3	Descriptive study No control group Enrollment method: not random	Perioperative treatment: none	Not evaluated	Not evaluated	Highest HtrA1 levels found in patients with stage I, II and IV_S_ vs. stage III-IV disease (P=0.003)^a^
Lorenzi *et al*, 2013 (43)	Bladder	152	Healthy individuals: Male, 6 Female, 32 Mean age, 65±5.5 Patients with urothelial bladder cancer:: Male, 50 Female,18 Mean age, 68.2±7.0 Patients with bacterial cystitis: Male, 4 Female, 12 Mean age, 59.1±11.8	Diagnostic study with analysis of of sensibility and specificity for urinary HtrA1 Control: healthy control Enrollment method: consecutive	Perioperative treatment: not declared	Lower expression of HtrA1 protein (autocatalytic form) in cancer than in normal-appearing tissue (P<0.001)	Not evaluated	Not evaluated
Lehner *et al*, 2013 (11)	Breast	131	Female only Age <50, 23 Age >50, 108	Longitudinal study Tissue selected with criteria (see text for detail) Survival analysis (see Table II) Enrollment method: not random	Unilateral cancer Perioperative treatment: none	Not evaluated	Not statistically significant	Low T (pT1–2) associated with higher HtrA1 mRNA levels compared with high T (pT3–4) (P=0.025)^b^ No correlation with lymph node status (P=0.439) (see text for details)
Yu *et al*, 2012 (44)	Esophagus	63	Male, 50 Female, 13 Mean age, 73.4 Range, 45–79	Control: normal-looking tissue. Enrollment method: probably consecutive	Perioperative treatment: none	Significantly lower HtrA1 levels in cancer than in normal-appearing tissue; mRNA (P=0.004) expression and protein expression (P<0.05)	Stage of differentiation also correlates with HtrA1 mRNA levels for mRNA P=0.024) than protein expression (P<0.05)	Pathological stage correlates with mRNA expression as well as protein expression (P=0.013)
Xia *et al*, 2012 (45)	Esophagus	115	Male, 71 Female, 44 Age <60, 5 Age >60, 63	Longitudinal study Survival analysis (see Table II) Enrollment method: not reported	Perioperative treatment: not reported	Significantly lower HtrA1 mRNA and protein in cancer specimens than in normal-appearing tissue (P<0.05)	Not statistically significant	Low HtrA1 levels, as mRNA or protein, are associated with TNM stage and lymph node metastases (P<0.05)
Catalano *et al*, 2010 (12)	Stomach	80	Male, 51, Female, 29 Mean age, 64 Range, 32–82	Longitudinal study, Survival analysis (see Table II) Enrollment method: consecutive	Patients with recurrent or metastatic cancer subjected to first-line chemotherapy (5-FU and cisplatin)	Not evaluated	Not evaluated	Not statistically significant
Zhu *et al*, 2010 (10)	Liver	50	Male, 42 Mean age, 52.43±9.94 Female, 8 Mean age, 50.39±14.12	Longitudinal study Control: normal- appearing tissue Survival analysis (see Table II) Enrollment method: probably consecutive	Perioperative treatment: none	Expression of HtrA1 lower in HCC than in normal- appearing specimens (P=0.045)	HtrA1 expression and staining score related to histological grade^c^ (grade I-II higher than grade III-IV) (P=0.036)	TNM or clinical stage not evaluated No correlation with tumor and size, metastasis (see text for detail)
Mullany *et al*, 2010 (46)	Endometrium	184	Mean age, 66.1±11.3	Longitudinal study Survival analysis (see Table II) Enrollment method: random		Not evaluated	Significantly lower HtrA1 levels in high-grade (3) than in low-grade (1–2) tumors (P=0.016)	Not statistically significant
Narkiewicx *et al*, 2008 (47)	Endometrium	124	Age not reported	Control: healthy subjects.^d^ Enrollment method: not reported	Perioperative treatment: Not reported	Significantly lower HtrA1 mRNA and protein levels in cancer than in control specimens (P<0.001)	Not evaluated	Not statistically significant
Bowden *et al*, 2006 (48)	Endometrium	33	Age not reported	Control: healthy subjects.^d^ Enrollment method: not reported	Perioperative treatment: not reported	Significantly lower HtrA1 mRNA in tumors of all grades than in normal endometrium (P<0.001)	Lowest levels of HtrA1 mRNA from G2 to G3 (P<0.01)	Not evaluated
Zurawa- Janicka *et al*, 2012 (49)	Thyroid	40	Not reported	Control: healthy subjects. Specimens from 40 patients: 20 with benign lesions and 20 with cancer Enrollment method: not reported	Perioperative treatment: not reported	Not statistically significant	Not evaluated Evaluation of HtrA1 levels between two different histology (See text for detail)	Not evaluated
Baldi *et al*, 2008 (9)	Pleura (mesothelioma)	70	Female, 29 Male, 41 Median age, 65 Range, 45–81	Longitudinal study Survival analysis (see Table II) Enrollment method: probably consecutive	All patients were treated with radiotherapy or chemotherapy 44 patients were treated with cyto- reductive surgery	Not evaluated	Not evaluated	Not evaluated
Narkiewicz *et al*, 2008 (50)	Ovary	98	Not reported	Study with five different groups^e^ Enrollment method: not reported	Perioperative treatment: not reported	Significant decrease in HtrA1 mRNA levels was observed in the malignant tumor tissue group compared to the normal ovarian tissue (P<0.001). Not significant for protein (see text for details)	Not statistically significant	Not statistically significant
Esposito *et al*, 2006 (51)	Lung	99	Median age, 58 Male, 73, Female, 13	No control group Enrolment method: consecutive	Perioperative treatment: none	Not evaluated	Non-significant differences between histological grades (see text for detail)	Not statistically significant
Baldi *et al*, 2002 (8)	Skin (melanoma)	11	Not reported	No control group Enrolment method: not reported	Perioperative treatment: not reported	Not evaluated	Not evaluated	Correlation with T (P=0.002)

a NB International Stage System;

b Bloom-Richardson criteria;

c Edmondson and Steiner’s criteria;

d without malignancy;

e five groups: 19 normal tissues, 20 benign tumors, 7 borderline tumors, 8 Krukenberg tumors, 44 carcinomas.

**Table II:** Observational longitudinal studies.

Author, year (ref.) cancer type	Aim	Follow-up	Cohort Overall survival (OS)	Disease-free survival (DFS)	Hazard ratio (HR)
Lehnert *et al*, 2013 (11) Breast	Evaluation of HtrA1 mRNA expression on patient outcome Two groups selected with a cut-off of 48	60 months 120 months	60 months: 85±3.2% SE 120 months: 66±4.9% SE	60 months: 75±4.0% SE 120 months: 54.5±5.2% SE	OS: HR 0.45 (95% CI 0.23–0.90) P=0.023 DFS: HR 0.55 (95% CI 0.32–0.94) P=0.028 Lymph node-positive groups: OS: HR 0.21 (95% CI 0.07–0.63) P=0.006 DFS: HR 0.29 (95% CI 0.13–0.65) P=0.003
	(see Table III for Laboratory techniques)				Therapy groups: (none, endocrine, chemotherapy) OS: HR 0.23 (95% CI 0.07–0.72) P=0.0012 DFS: HR 0.29 (95% CI 0.13–0.67) P=0.004
Xia *et al*, 2013 (45) Esophagus	Correlation between HtrA1 levels and survival	60 months	The overall cohort survival, disease-free survival and HR: were not reported. Reported date included: HtrA1 mRNA-positive: 13 of 39 patients died (33.3%) HtrA1 mRNA-negative: 56 of 76 patients died (73.7%) (P<0.05)		
Catalano *et al*, 2011 (12) Stomach	Determine HtrA1 expression as predictor of chemoresponse in patients with advanced gastric cancer Two groups high/medium expression vs. low expression (see Table III for laboratory techniques)	60 months	17.0 months: high/medium HtrA1 expression 9.5 months: low HtrA1 expression	Not reported, they evaluated time to progression High and medium HtrA1 expression compared with low: 0.52 (95% CI 0.29–0.93) P=0.027	OS: HR 0.55 (95% CI 0.32–0.96) P=0.037
Mullany *et al*, 2011 (46)	Dichotomized into 2 groups high intensiy score vs. low	200 months	Not reported	No significant relationship between high-medium expression of HtrA1 and survival	
Endometrium Zhu *et al*, 2010 (10) Liver	Dichotomized into 2 groups high intensity score vs. low	72 months	HtrA1 (+++/++) survival rate 46%, median survival 35.5 months HtrA1 (+) survival rate 26%, median survival 15.6 months Cohort OS, DFS and HR not reported		
Baldi *et al*, 2008 (9) Pleural mesothelioma	Analyzed the potential prognostic value of expression of HtrA1 Three class of HtrA1 expression: low medium high	40 months	HtrA1 (+): median survival time (months), 6.0 (95% CI 4.46–7.51) HtrA1 (++): median survival time (months), 16.0 (95% CI 12.54–19.45) HtrA1 (+++): median survival time (months), 24.0 (95% CI 20.50–27.49) P<0.0001	Not reported	HtrA1 (+): HR 1 (reference category) HtrA1 (++): HR 0.65 (95% CI 0.348–0.876) HtrA1 (+++): HR 0.26 (95% CI 0.122–0.454) P<0.001

**Table III:** Laboratory methods used in the studies of HtrA1 in cancer.

Authors/ref.	Involved organ	Methods		mRNA		Protein	
		*In vitro*	*In vivo*	ISH	PCR	IHC	WB
D’Angelo *et al* (42)	Nerve cells		√			√	√
Lorenzi *et al* (43)	Bladder	√	√		√	√	√
Lehner *et al* (11)	Breast	√	√		√		
Yu 2012 *et al* (44)	Esophagus	√	√		√	√	√
Xia 2013 *et al* (45)	Esophagus	√	√	√	√	√	√
Catalano *et al* (12)	Stomach		√			√	
Zhu *et al* (10)	Liver		√			√	
Mullany *et al* (46)	Endometrium	√	√			√	
Narkiewicz *et al* (47)	Endometrium		√		√		√
Bowden *et al* (48)	Endometrium		√		√	√	√
Zurawa *et al* (49)	Thyroid		√				√
Baldi *et al* (9)	Pleural mesothelioma	√	√			√	√
Narkiewicz *et al* (50)	Ovary		√		√		√
Esposito *et al* (51)	Lung		√			√	
Baldi *et al* (8)	Skin cells (Malignant melanoma)	√	√			√	

ISH, *in situ* hybridization; PCR, polymerase chain reaction; IHC, immunohistochemistry; WB, western blotting.

**Table IV:** HtrA1 as a potential tumor marker.

Cancer type	Early marker	Prognostic marker	Tumor marker	Author/ref.
Neuroblastoma				
Ganglioneuroblastoma			√	D’Angelo *et al* (42)
Bladder	√		√	Lorenzi *et al* (43)
Breast		√	√	Lehner *et al* (11)
Esophagus			√	Yu *et al* (44)
Esophagus		√	√	Xia *et al* (45)
Stomach		√	√	Catalano *et al* (12)
Liver		√	√	Zhu *et al* (10)
Endometrial		√	√	Mullany (46)
Endometrial			√	Narkiewicz *et al* (47)
Endometrial			√	Bowden (48)
Thyroid			√	Zurawa *et al* (49)
Pleural mesothelioma		√	√	Baldi *et al* (9)
Ovary			√	Narkiewicz *et al* (50)
Lung			√	Esposito *et al* (51)
Malignant melanoma			√	Baldi *et al* (8)
